# Pediatric sellar teratoma – Case report and review of the literature

**DOI:** 10.1007/s00381-024-06296-w

**Published:** 2024-01-26

**Authors:** Katja Kürner, Ladina Greuter, Michel Roethlisberger, Yves Brand, Stephan Frank, Raphael Guzman, Jehuda Soleman

**Affiliations:** 1 Department of Neurosurgery, University Hospital of Basel, Spitalstrasse 21, 4031 Basel, Switzerland; 2 Faculty of Medicine, University of Basel, Basel, Switzerland; 3 Department of Otorhinolaryngology, Cantonal Hospital Graubünden, Chur, Switzerland; 4 Department of Pathology, Division of Neuropathology, University Hospital of Basel, Basel, Switzerland; 5 Division of Pediatric Neurosurgery, University Children’s Hospital of Basel, Basel, Switzerland

**Keywords:** Sellar teratoma, Pediatric brain tumor, Non-germinomatous germ cell tumor, Transsphenoidal endoscopy

## Abstract

**Background:**

Intracranial teratoma represents a rare neoplasm, occurring predominantly during childhood. Characteristic symptoms depend on the location but are mainly hydrocephalus, visual disturbances, hypopituitarism, and diabetes insipidus. Initial diagnosis can be challenging due to similar radiological features in both teratomas and other lesions such as craniopharyngiomas. Gross total resection is recommended if feasible and associated with a good prognosis.

**Case description:**

A 10-year-old girl presented with newly diagnosed growth retardation, fatigue, cephalgia and bilateral hemianopia. Further laboratory analysis confirmed central hypothyroidism and hypercortisolism. Cranial magnetic resonance imaging showed a cystic space-occupying lesion in the sellar and suprasellar compartment with compression of the optic chiasm without hydrocephalus present, suspicious of craniopharyngioma. Subsequently, an endonasal endoscopic transsphenoidal near-total tumor resection with decompression of the optic chiasm was performed. During postoperative recovery the patient developed transient diabetes insipidus, the bilateral hemianopia remained unchanged. The patient could be discharged in a stable condition, while hormone replacement for multiple pituitary hormone deficiency was required. Surprisingly, histopathology revealed conspicuous areas of skin with formation of hairs and squamous epithelia, compatible with a mature teratoma.

**Conclusions:**

We present an extremely rare case of pediatric sellar teratoma originating from the pituitary gland and a review of literature focusing on the variation in presentation and treatment. Sellar teratomas are often mistaken for craniopharyngioma due to their similar radiographic appearances. However, the primary goal of treatment for both pathologies is to decompress eloquent surrounding structures such as the optic tract, and if applicable, resolution of hydrocephalus while avoiding damage to the pituitary stalk and especially the hypothalamic structures. If feasible, the aim of surgery should be gross total resection.

**Supplementary Information:**

The online version contains supplementary material available at 10.1007/s00381-024-06296-w.

## Introduction

Intracranial germ cell tumors (ICGCT) are rare and account for approximately 3% of all central nervous system (CNS) tumors in children and young adults and they rarely occur after the 4th decade of life [1–3]. The incidence of pediatric ICGCTs varies considerably between different regions. The incidence in Europe and the United States is around 0.5–3% of all CNS tumors, while in Asia, especially Japan, the incidence ranges up to 8–14% [4–6]. The genetic factors influencing this difference are not fully elucidated [7].

ICGCTs mostly arise in the midline, either in the sellar or pineal region. Based on the WHO classification of CNS tumors, intracranial germ cell tumors comprise a heterogeneous group of neoplasms, divided into germinomas and non-germinomatous germ cell tumors (NGGCT) [1, 4, 8]. While germinomas account for up to 50–70% of ICGCTs, NGGCTs are further subdivided into embryonal carcinomas, yolk sac tumors, choriocarcinomas, mature teratomas, immature teratomas, and mixed germ cell tumors [4, 10]. Mature teratomas consist of fully differentiated elements derived from one or all three germ cell layers, while immature teratoma contains incompletely differentiated tissue [9]. Despite the fact that teratoma may manifest anywhere in the midline, most occur in the pineal region and only few cases of pediatric sellar teratomas are described in the literature [6, 10–20].

In this article we present a case report of a ten-year-old girl presenting with a suprasellar mature teratoma and provide a literature review thereof focusing on the treatment options.

## Case description

**Fig. 1:**
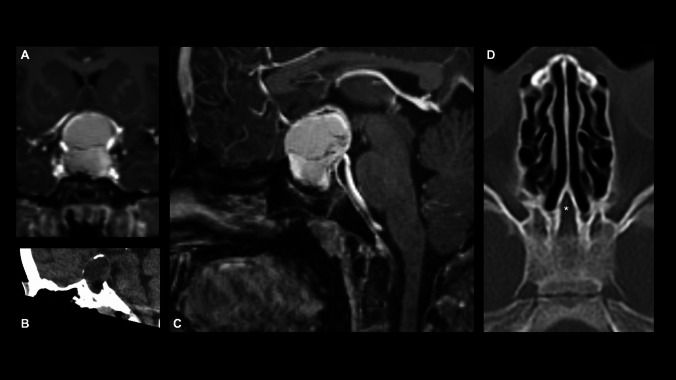
**A** Coronal T1-weighted magnetic resonance imaging (MRI). **B** Sagittal soft tissue-weighted computed tomography (CT). **C** Sagittal T1-weighted MRI demonstrating a 3 × 3 × 2.5 cm cystic space-occupying partially calcified lesion in the sellar and suprasellar compartment with compression of the optic chiasm, highly susceptive of a craniopharyngioma (CP). **D** Axial bone-weighted CT demonstrating a non-pneumatized sphenoid bone with a hypo intense bone-marrow (*asterisk*)

**Fig. 2:**
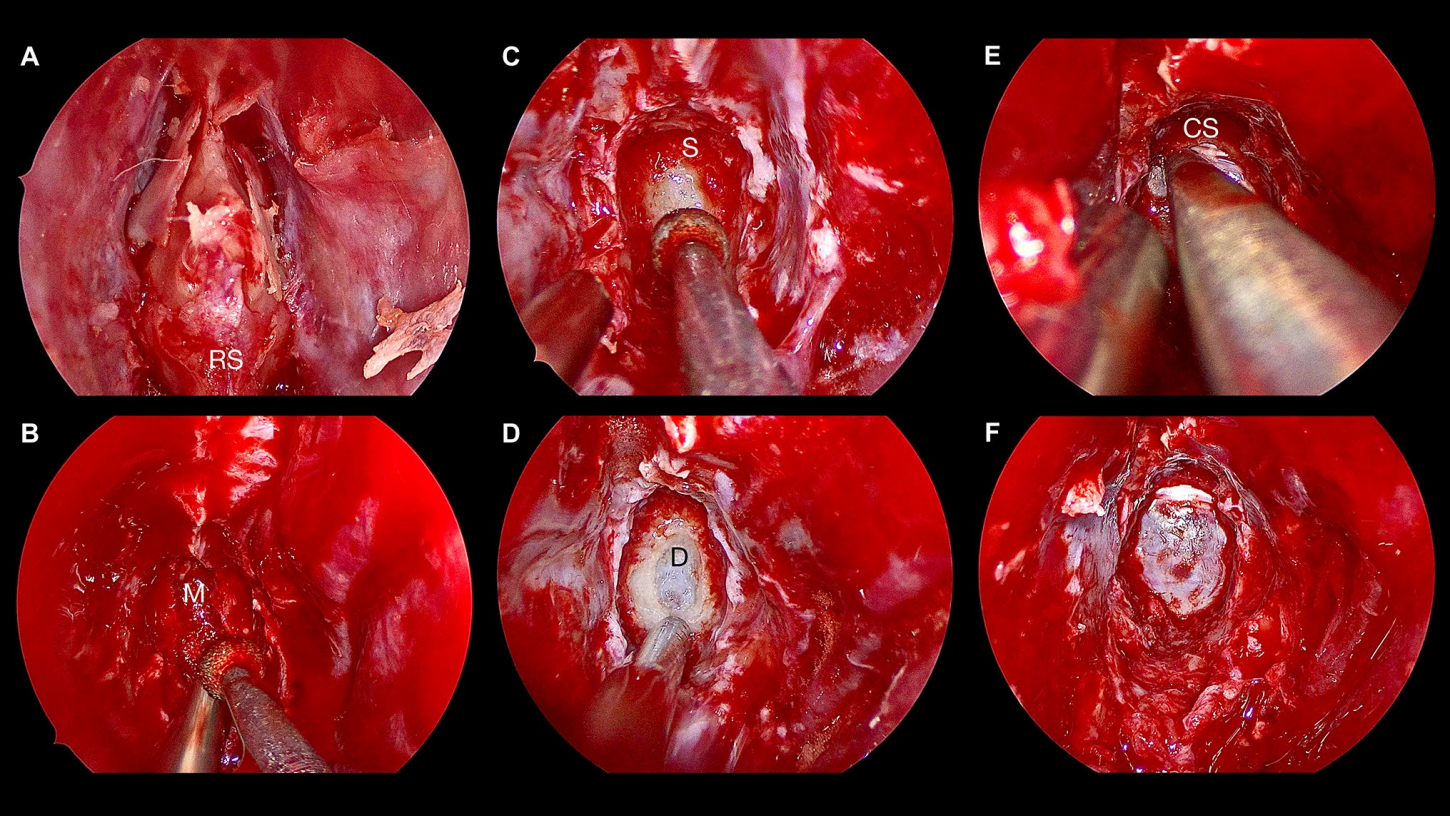
Endonasal endoscopic approach in a conchal type sphenoid sinus for a sellar and suprasellar cystic lesion. **A** Binostril approach and exposure of the rostrum sphenoidale [RS]. **B** Removal of the spongious and fatty sphenoidal bone marrow [M]. **C** Change of the color and consistency indicates exposition of the harder sellar bone [S]. **D** Trans-sellar exposure of the sellar dura [D]. **E** Trans-chiasmatic sulcus superior extension. **F** Complete osteodural trans-sellar trans-chiasmatic sulcus exposure prior dural opening

**Fig. 3:**
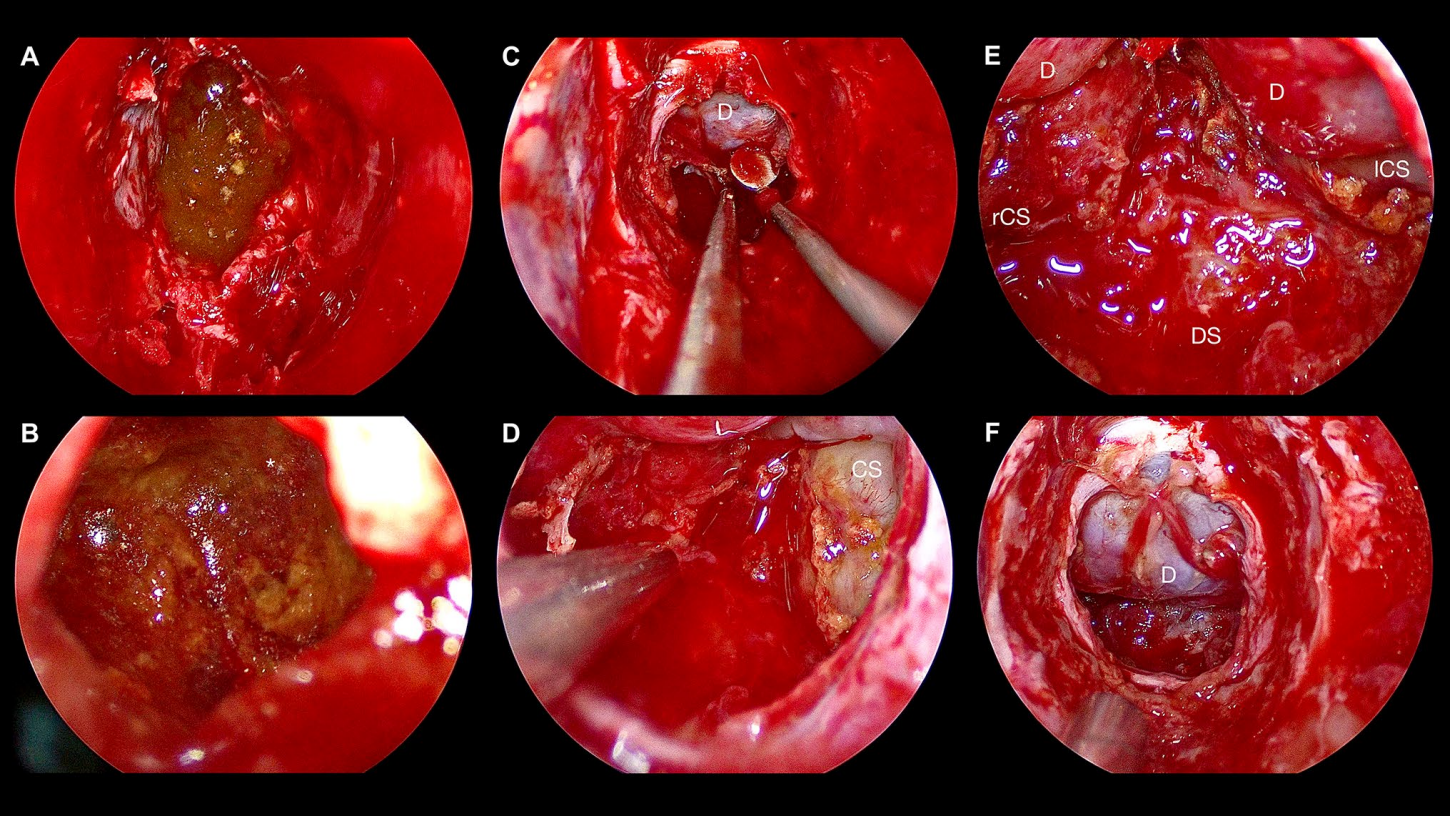
Endonasal endoscopic approach in a conchal type sphenoid sinus for a sellar and suprasellar cystic lesion. **A** Extrusion of crystals [*] and brownish “oily” cystic fluid. **B** Close-up inspection of the cystic content reveals solid component consisting of crystals [*] and debris. **C** Dissection of the suprasellar tumor cyst from the partially descending sellar diaphragm [D]. **D** Dissection of the tumor cyst from the left medial cavernous sinus wall [CS]. **E** Incomplete diaphragmal [D] descent indicating remaining suprasellar tumor components. Dissection from the left medial cavernous sinus wall [lCS] and the dorsum sellae [DS] was possible, while the cyst was very adherent to the right medial cavernous sinus wall [rCS]. **F** Symmetrical diaphragmal [D] descent at the end of the surgery prior skull base defect closure

**Fig. 4:**
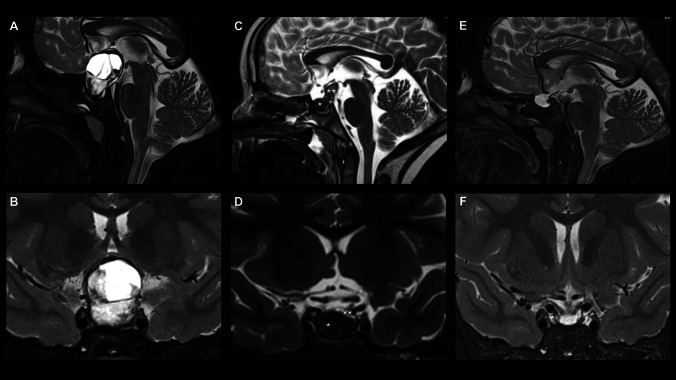
Radiological course. **A** Preoperative sagittal and **B** coronal T2-weighted MRI of the cystic sellar and suprasellar infantile mature teratoma. **C** Immediate postoperative sagittal and **D** coronal T2-weighted MRI demonstrating skull base defect reconstruction material within the sphenoid cavity and the sella turcica [*], along with a decompressed and anatomically located optic chiasm and remnant solid tumor supero-posteriorly [**]. **E** 1-year postoperative sagittal and **F** coronal T2-weighted MRI demonstrating resorption of the intrasellar reconstruction material along with volumetric regression and descent of the solid tumor remnant along the pituitary stalk into the dorsal aspect of the sella turcica. The optic nerves and chiasm descent into the sellar region and remains decompressed and anatomically preserved

**Fig. 5:**
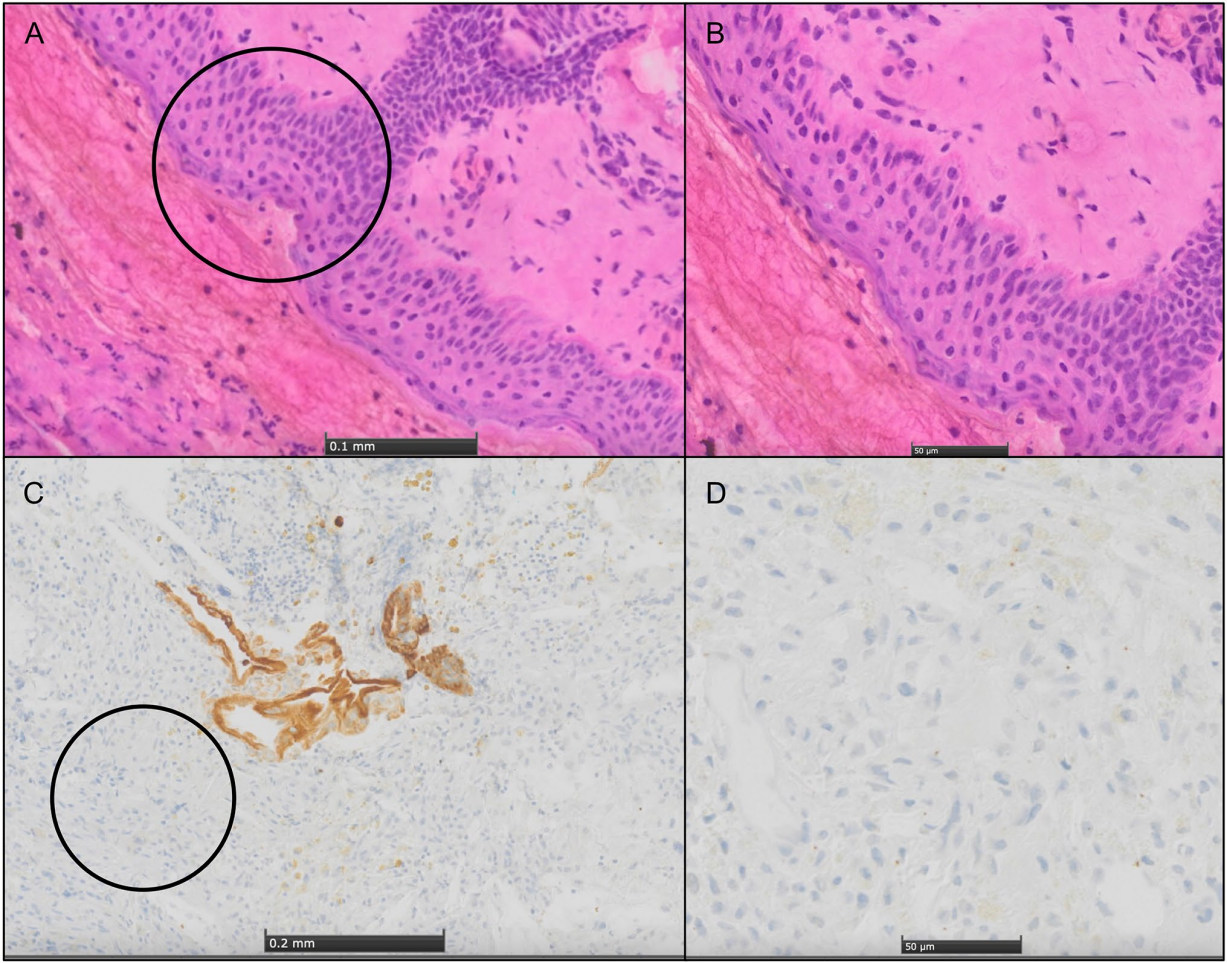
Histopathology of the sellar tumor. **A** Hematoxylin-Eosin (HE) stain 10× magnified showing areas of skin formation, circle indicates the area which is **B** zoomed into with a magnification of 20x. **C** Pan-Cytokeratin (CK22) stain, 5× magnified, proving epithelial cell presence and circle indicates the area which is **D** zoomed into with a magnification of 20×

A ten-year-old girl with a history of cystic fibrosis (CF) with gastrointestinal and pulmonary involvement, presented to her pediatrician with newly diagnosed growth retardation, fatigue and frequent headache. An arginine growth hormone-releasing hormone test confirmed growth hormone (GH) deficiency, and after further laboratory analysis, a central hypothyroidism and hypercortisolism was diagnosed. Substitutional therapy with levothyroxine and hydrocortisone was initiated, which improved the patient’s headache and fatigue. Ophthalmologic examination revealed bitemporal hemianopia. Further work-up with magnetic resonance imaging (MRI) revealed a 3 × 3 × 2.5 cm cystic space-occupying lesion in the sellar and suprasellar compartment with compression of the optic chiasm, with partial calcifications in computed tomography (CT), highly suspicious of a craniopharyngioma (CP) (Fig. 1A–C). Hydrocephalus was not present at the time of presentation. Due to the compression of the chiasm causing clinical hemianopia, the indication for surgical decompression was given. We weighed the possibilities of a neuroendoscopic transventricular (NET) cyst fenestration and partial tumor resection versus an endonasal endoscopic approach (EEA) or an open transcranial approach. The advantage for the transventricular neuroendoscopic approach is its minimal-invasive nature and straight-forward decompression of the cyst, which is causing compression of the optic chiasm. However, the patient presented with very small ventricles, making neuroendoscopy cumbersome, and the neuroendoscopic approach would only allow a partial tumor resection. EEA shares the advantage of being minimal-invasive. However, due to her age and the concomitant diagnosis of CF, known to be associated with hypoplasia and markedly reduced pneumatization of the paranasal sinuses, a non-pneumatized (conchal type) sphenoid was present (Fig. 1D), making the AAE more challenging [21]. In addition, gross total resection (GTR) was not considered achievable through an EEA, due to a supra-chiasmatic tumor extension. Still, both an NET or EEA seemed superior to an open approach (e.g. subfrontal or interhemispheric) given the invasiveness and associated morbidity of such approaches, while similar to the EEA and NET approach, GTR would most probably not be achieved either [22–26]. We ultimately decided to perform an EEA together with our colleagues from ENT. The conchal configuration of the sphenoid sinus required meticulous drilling of squamous intrasphenoidal bone, exposure of the harder sellar bone, and a superior trans-chiasmatic sulcus extension to achieve satisfactory exposure of the suprasellar tumor cyst (Fig. 2). Intraoperatively, crystals and cystic fluid, suspicious of CP, were drained from the cyst, and the cyst was dissected from the cavernous sinus walls, the sellar diaphragm, and the dorsum sellae without risking injury of adjacent structures. At the end of the operation, a symmetrical diaphragmal descent was achieved as indirect sign for the decompression of the optic chiasm (Fig. 3, Supplementary Video 1). Postoperative MRI showed the expected near total tumor removal. While the cyst was completely drained, tumor remnants extending posteriorly to the superiorly displaced chiasm remained as expected (Fig. 4). Postoperatively, the patient developed diabetes insipidus (DI) for which she received desmopressin under the supervision of the pediatric endocrinologists. During her inpatient stay, she recovered from her DI with stable sodium levels but required vasopressin substitution. Overall recovery was good, while the hemianopia persisted. No signs of rhinorrhea resulting from cerebrospinal fluid (CSF) fistula were noted. We were able to discharge the patient to her home 11 days after surgery. Unexpectedly, the histopathologic analysis found conspicuous areas of skin with formation of hairs and squamous epithelia, compatible with a mature teratoma (Fig. 5). The cytokeratin staining was positive for epithelial cells consistent with the finding in a mature teratoma (Fig. 5B, C). These findings led to the diagnosis of a rare case of infantile mature teratoma originating from the sellar region After discussion in our interdisciplinary pediatric neuro-tumor-board, no further treatments (e.g. chemotherapy, radiation therapy) were indicated and a clinical and radiological follow up was initiated.

At her first postoperative follow-up appointment after 6 weeks the patient was back in school, without any complaints. The hemianopia remained unchanged, and cortisol, vasopressin and thyroxin substitution was still required one year postoperatively. She was also started on growth hormone (GH) replacement therapy 6 months postoperatively. MRI follow-up one year after surgery showed stable appearances without any sign of progression (Fig. 4).

## Discussion

Although intracranial teratomas account for only about 0.5% of all intracranial tumors, they represent a highly relevant differential diagnosis for pediatric midline tumors [27]. The literature on pediatric sellar teratomas is scarce, and to our knowledge only 4 cases of mature sellar teratoma have been reported to date (Table 1) [6, 10–20].

**Table 1:** Overview of previously published pediatric sellar teratoma case reports

**Author**	**Year**	**Gender**	**Age (years)**	**Endocrine deficits pre op (DI, Hypopituitarism)**	**Histopathological Diagnosis**	**Treatment**	**Surgical Approach**	**Extent of Resection**	**Follow up**
**Fults and Kelly **[10]	1983	F	15	no	atypical teratoma	n.a.^a^	n.a.	n.a.	n.a.
**Lee et al. **[11]	1995	M	5	yes	immature teratoma	1. radiotherapy, 2. surgery	Craniotomy what kind of craniotomy? Pterional, subfrontal?	Subtotal tumor excision	n.a.
**Cho et al. **[12]	1997	M	8	yes	intrasellar mixed GCT^b^	1. surgery, 2. adjuvant chemotherapy, 3. radiotherapy, 4. chemotherapy	Transsphenoidal approach	Subtotal tumor excision	6 months: Good, no disorders
**Ikeda et al. **[13]	1998	M	10	yes	Germinoma and mature teratoma	surgery	Occipital transtentorial approach	Total tumor excision	Neurologic deficits unchanged (homonymous hemianopsia, left hemiparesis), 2. De novo metachronous neoplasm after 8 years (germinoma)
**Araki et al. **[20]	2000	M	0.25	no	mature teratoma	surgery	n.a.	n.a.	Obesity, high serum insulin
**Yagi et al. **[14]	2004	F	16	yes	immature teratoma and mature teratoma	1. debulking followed by chemotherapy, 2. debulking followed by radtiotherapy, 3. radical surgery followed by chemotherapy	Interhemispheric and transcallosal intraventricular approach	n.a.	n.a.
**Chang et al. **[15]	2008	F	5	yes	Mixed GCT	1. debulking, 2. chemotherapy	n.a.	n.a.	n.a.
**Vendrell et al. **[16]	2010	M	1.5	no	Mature Teratoma	Debulking	Transsphenoidal approach	Debulking	6 months: vision loss of the left eye
**Kim et al. **[17]	2010	M	17	yes	Mature teratoma	1. surgery, 2. chemotherapy, 3. radiotherapy	Transsphenoidal approach	n.a.	n.a.
**Tsoukalas et al. **[6]	2013	F	17	yes	GCT with teratoma and CP^c^	1. chemotherapy, 2. autologous stem cell therapy, 3. radiotherapy	-	Persisting tumor mass (elements of CP)	3 months: patient deceased
**Chiloiro **[18]	2015	F	6	yes	Mature teratoma	1. radiotherapy (susptected germinoma), 2. Surgery, 3. rhGH and estrogen-progestin replacement therapy	n.a.	resection	4 years: hypopituitarism, 12 years later: 2. Neoplasia (atypical meningioma)
**Yoneoka et al. **[19]	2018	M	14	yes	Immature teratoma with germinoma	1. surgery, 2. chemotherapy	Subfrontal via lamina terminalis and extended transsphenoidal approach	GTR^d^ in 2 stages	3 years: complete remission
**Summary:** Reports found: 12	1983–2018	Male: 7, (58.3%) Female: 5, (41.7%)	Mean Age: 9.6 years	Endocrine dysfunction: Yes: 9, (75%), No: 3, (25%)	Atypical teratoma: 1, (8.3%), Mature teratoma: 4, (33.3%) Immature teratoma: 1, (8.3%) Mixed GCT: 6, (50%)	Treatment: Surgery alone: 3, (25%), Surgery+chemotherapy: 2, (16.7%), Surgery+radiotherapy: 1, (8.3%), Surgery+chemotherapy+radiotherapy: 3, (25%) Others: 2, (16.7%), n.a.: 1, (8.3%)	In case of surgery (9): Transsphenoidal approach: 4, (44.4%) Occipital transtentorial: 1, (11.1% Interhemispheric/transcallosal/intraventricular: 1, (11.1%), n.a.: 3, (33.3%)	In case of surgery (9): Subtotal tumor excision: 4, (44.4%), Total tumor excision: 3, (33.3%), n.a.: 2, (22.2%)	Complete remission without deficits: 2, (16.7%, Complete remission with remaining deficits: 2, (16.7%), Neoplasia after treatment: 2, (16.7%) Patient deceased: 1, (8.3%), n.a.: 5, (41.7%)

^a^n.a. Information not available

^b^*GCT* Germ Cell Tumor

^c^*CP* Craniopharyngioma

^d^*GTR* Gross Total Resection

Symptoms are often unspecific and include headaches and signs of increased intracranial pressure in case of hydrocephalus, while visual impairment, mainly bitemporal hemianopia, panhypopituitarism and DI can be found at a more chronical stage of the disease [12, 27, 28]. In our case, multiple pituitary hormone deficiencies were diagnosed due to short stature with delayed skeletal age. A more thorough testing revealed central hypothyroidism and hypocortisolism, for which substitution therapy was initiated preoperatively. According to Esfahani et al., pediatric suprasellar germ cell tumors (GCT) usually present primarily with DI, while other symptoms due to compression of the anterior pituitary are hypocortisolism and hypothyroidism [12, 27]. The symptom complex of frequent headaches, bitemporal hemianopia, growth retardation, and DI strongly suggests the differential diagnosis of a sellar tumor, which is most commonly a CP but could be any other midline pathology in this region [30, 31]. Based on the MRI findings in our case, which showed a partially cystic and partially solid lesion (Fig. 1), the diagnosis of a CP was further substantiated. It has been previously reported that NGGCT in the sellar region can mimic CP due to their similar clinical and radiographic characteristics [15, 31–33]. Both tumors have radiographic hallmarks of mixed density, usually with cystic and solid components, while for mature teratomas an occasional finding of teeth, fat, hair, or calcifications is possible [17, 20, 27, 34–37]. Classically, mature teratomas contain cells of all ecto-, meso- and endodermal layers, however, not all teratomas exhibit all three tissue layers, which can make their diagnosis more challenging. Teratoma arise from the midline structures and as in most intracranial GCT they tend to occur in either the pineal region or more rarely suprasellar [38, 39]. In some cases teeth or fat tissue can be visualized on a preoperative scan, which can facilitate diagnosis, however, in most reported cases, the teratoma mimicked either a CP or a Rathke’s cleft cyst [16, 38].

Craniopharyngiomas on the other hand, arise from the Rathke’s cleft epithelium, and hence also occur in the midline. They are observed to have a bimodal distribution occurring in children and in people in their 5-6th decades of life, while teratomas mainly occur in children and young adults [40].

Intracranial NGGCTs can show elevated serum and CSF markers like alpha-fetoprotein (AFP), human beta-chorionic gonadotropin (ß-HCG) and placental alkaline phosphatase (PLAP), however, they are not typically elevated in mature teratomas [29, 41–43]. In our case, tumor markers were not measured, as we suspected a CP in the first place. Therefore, the diagnosis was ultimately achieved by histopathological analysis. Huang et al. recently reported that CCND2, RB1, and PRDM14 are involved in the cellular and molecular pathogenesis of IGCTs. Moreover, they suggest KIT/RAS and AKT1/mTOR signaling pathways as possible therapeutic targets [44].

In most cases of space-occupying sellar tumors with neurological deficits surgical therapy is indicated to decompress the nervous structures but also to reach an ultimate histopathological diagnosis [27, 41, 45].

Tumors originating from the sella can be reached either endoscopically through an EEA, NET, or by open pterional, interhemispheric, transfrontal-transcortical with or without a transchoroidal window, or subfrontal craniotomy, depending on the tumor extension. In young children, the transsphenoidal approach can be more challenging due to incomplete pneumatization of the sphenoid sinus, which does not usually begin until after the third year of life and is only fully developed at about 15 years of age, increasing the likelihood of intraoperative hemorrhage [22]. In addition, transsphenoidal approaches are limited for suprachiasmatic tumor extension, or lateral tumor extension, given the proximity to the carotid arteries [23, 24]. Moreover, a study showed that CSF fistulas occurred at a higher rate in EEA compared to open transcranial approaches for pediatric sellar lesions [24]. For tailored extended EEA approaches with GTR or near-total resection strategies, experience in dealing with graded repair for large skull base defects, harboring a relevant risk for high-flow CSF-leaks, is mandatory and should only be performed by experienced skull base neurosurgeons and ENT surgeons in an interdisciplinary setup [46, 47]. As our patient did not show any intraoperative CSF flow, along with a symmetrical descent of the diaphragm, a defect reconstruction using only resorbable hemostyptic materials without the need of an abdominal fat graft or pedicled vascularized flap was justified (Fig. 3,Video 1). Overall, EEA is appropriate for resection of pediatric tumors of the sellar region and shows good results, if done in experienced centers and interdisciplinary manner including pediatric neurosurgeons, skull-base surgeons and ENT surgeons [25, 48]. Another endoscopic approach is the NET for tumor cyst fenestration via the 3rd ventricle, which is also a minimal invasive method, however an extensive resection of the tumor can hardly be achieved due to limited hemostatic control and access route. Open surgery is the traditional approach and feasible for large tumors, yet the localization of the optic nerves, the carotid arteries and the hypothalamus must be carefully considered preoperatively. Further, open resection carries additional risk of morbidity due to brain retraction potentially causing seizures, hemorrhage, or ischemia [24]. In all of these approaches, it is of paramount importance that the hypothalamus is not injured during surgery, and in case of hypothalamic tumor invasion, GTR should not be the main aim of surgery but rather an intended near-total resection, as in our case (Fig. 3,Video 1) [25, 49]. In children, hypothalamic injury results in hyperphagia, obesity, neurocognitive deficits, and lower quality of life and should be avoided at all costs, especially before puberty, independent of the underlying diagnosis [50–52]. Other common complications after sellar/suprasellar surgery include diabetes insipidus (DI), endocrine disturbances or CSF leak [36, 53, 54]. Our patient suffered from postoperative DI, requiring long-term treatment with desmopressin. A recent meta-analysis by Lee et al. concluded that approximately one quarter of all patients developed new permanent DI after surgery for pediatric sellar and suprasellar lesions [54].

Children diagnosed with GH deficiency need to be closely monitored to avoid any disproportionate growth or growth retardation. According to a recent consensus statement on the safety of hormone replacement in survivors of cancer and intracranial including pituitary tumors, the timing of initiation of GH therapy after surgery is multifactorial and should be decided individually and discussed thoroughly with all parties involved.

Radiological evidence of tumor stability is always a prerequisite for start of therapy. However, in children with radiologically stable craniopharyngiomas showing significant growth and metabolic disturbances, this may be considered as early as 3 months after the end of tumor therapy. Furthermore, based on current scientific evidence, there appears to be no association between GH replacement and increased cancer-related mortality in childhood cancer survivors [55]. Our patient did not only suffer from hormonal deficiency but also had a history of CF. While the literature often mentions an association between CF and various types of cancer, mainly gastrointestinal tumors, but also skin, lung, hematological and breast cancers, there is no evidence of an association between CF and brain tumors or cystic teratomas in general [56–68].

The recurrence rate of mature teratoma after GTR is low and long-term outcome is excellent with a reported 10-year survival rate of 93% [27, 69, 70]. Hence, GTR is considered the gold standard treatment and mature teratomas represent the only entity of germ cell tumors that can be treated by resection alone [27, 69, 71]. Other germ cell tumors such as immature teratomas or mixed GCT teratomas, require a multistage treatment involving surgical resection, adjuvant or neoadjuvant chemo- and radiotherapy [27, 39, 70]. In case of postoperative tumor remnant of a mature teratoma, like in our case, current treatment recommendation is radiographic observation and in case of a progression either re-resection, if surgically feasible, or radiotherapy [72]. Some reports suggest that Gamma knife radiosurgery may even be considered superior to conventional radiotherapy in the recurrence treatment of mature teratomas minimizing the risk of pituitary dysfunction as well as of hypothalamic damage [73–75]. Regardless of the extent of resection, especially pediatric patients will be under radiographic surveillance for numerous years and often require life-long hormone substitution. The importance to follow these patients over years and the difficulty of continuing care for these patients during transition to adulthood has been discussed within the literature [76–84].

## Conclusion

To our knowledge, this is one of very few case reports describing a pediatric sellar mature teratoma. Initial diagnosis can be challenging due to similar radiological features with CP, which is more frequent in this age group. In case the tumor causes compression of the optic chiasm or hydrocephalus, surgery is warranted regardless of the diagnosis. Depending on the tumor extension (suprasellar, third ventricle, lateral of the carotids) the most suitable surgical approach should be chosen. Moreover, when considering EEA in younger children, the pneumatization patterns of the paranasal sinuses and adjacent skull base structures should be analyzed carefully, and only an experienced interdisciplinary team including pediatric and skull base neurosurgeons, ENT surgeons and pediatric endocrinologists should treat these patients. Mature teratomas have a good overall prognosis, however these children require long-term follow up within the setting of a pediatric interdisciplinary neuro-oncological clinic.

### Supplementary information

Below is the link to the electronic supplementary material.

Supplementary Video 1 Intra- and suprasellar cystic-solid infantile mature teratoma in a child with conchal type sphenoid sinus – Extended endonasal endoscopic transsphenoidal trans-sellar and chiasmatic-sulcus approach, subtotal tumor resection and skull base defect reconstruction (MP4 548083 KB)
